# Supplementary material for: Catch the star! Spatial information activates the manual motor system
Source: PLoS One. 2022 Jul 8;17(7):e0262510. doi: 10.1371/journal.pone.0262510 (PMC9269453; doi:10.1371/journal.pone.0262510)
Supplement: Attachment — Submitted filename: PONE-D-21-39874 - Review.pdf [file pone.0262510.s001.pdf]

REVIEW – Catch the star! Spatial information activates the manual motor system

The study aims to evaluate the relationship between the manual motor system and exogenous and endogenous attention, the latter associated with two different forms of processing.

It is a very interesting manuscript, well written, addressing a relevant subject, and seeking evidence of important connections between motor function, central processing of sensory and motor information and attentional response.

I only have a few considerations about it, described below by section.

Introduction

The introduction was very well organized, presenting the theoretical framework in a structured and direct manner. The citations are current and relevant, properly building the line of reasoning that culminates in the presentation of the objective of the study. The reasons for investigating the relationship of the manual motor system with endogenous attention through two experiments, however, were not presented, causing a break in the development of the introduction. In this sense, lines 339-345 should be part of the introduction to justify the decision to carry out two experiments involving endogenous attention.

Methods and Results

The decision to present each experiment individually facilitates understanding. However, some aspects of the Methods that are valid for two or three experiments could be grouped together at the beginning of this section or presented in a single Table to allow a better visualization of the details of each experiment, e. g. exclusion of participants, exclusion of trials, accuracy, 5000 permutations performed in the cluster permutation analysis. There is no reason to describe the dips as they will not be discussed at any time. If you believe that these data are particularly important, Figures 2, 5, and 7 can present them satisfactorily, thus bringing more fluidity to the reading of the text.

The supplementary material brings the images of the stars and arrows as they were presented to the participants. However, there is no image for experiment 3.

I was not able to open the *words.osexp* file, and the manuscript text does not provide information regarding the font, serif, and word style.

Furthermore, in experiments 1 and 2, the stimuli were presented in two colors, one of which was associated with the verbal task. However, in experiment 3, in addition to colors, there are two types of text (words and symbols). Thus, the data sample for each condition dropped from 90 in experiments 1 and 2 to 60 in experiment 3, the same experiment that had a 35% drop in sample size. What is the point of introducing symbols arrays in Experiment 3, in red and yellow, if these constructions did not contribute to the discussion and were not statistically analyzed?

In addition, the sample has participants with different levels of consistency in manuality. Did this feature have no impact on the results? Especially in relation to experiment 3, due to its smaller sample, what was the impact of the sample low consistency in manuality? What was the behavior of the left-handed portion of the sample regarding the results obtained? Wouldn't removing these participants from the sample imply more consistent results?

At various times, you classify your findings as close to significance. This is especially noteworthy in experiment 2, when a result close to significance reached $p=.1$. What is the threshold for considering a result close to or far from significance? Would considering these values as not significant significantly change your results? I believe that the text needs to be better worked on this point.

Although you present the conditional r and the marginal r of each calculation and experiment, you do not comment on the relative value of their different values throughout the study. Could you comment more on this?

Discussion

In the discussion, you say that the force increased for one hand and decreased for the other, as per the experiment. However, in the graphs, it is not possible to observe a drop in force in relation to the moments prior to the peak force zone, that is, there is not a valley and a peak occurring simultaneously when observing the tracing for each hand in Figures 2C and 5C. Figure 7C is quite confusing as to the line of each condition in the interaction between Hand and Word area.

Is there really a drop in the force exerted by the contralateral hand in experiments 1 and 2 and by the ipsilateral hand in experiment 3? Is it not possible that the increase in force is simply happening later, as appears to be represented in Figure 7C? Please explain more about this.

Similarly, the hypothesis regarding the grip force increasing of the contralateral hand in experiment 3 lacks further detail. The text seeks to explain that the inhibitory stimulus for the execution of the movement of the ipsilateral hand has a more pronounced effect in experiment 3 due to a greater semantic processing. However, what would lead to an increase of the grip force of the contralateral hand? Or is this increase just relative? I believe this is the most awaited part of the discussion, and, as it stands, it is not at the level of the work performed with so much labor and perfection described in this manuscript.

Figures

In the text, the caption of figure 7 states that the interaction area between Hand and Word occurs between 830-860 ms, but, from the figure, I believe it occurs between 230-260 ms.

The Figures 2C, 5C and 7C are very important to the manuscript and deserve a better treatment. The color code used for the interaction areas is confused with the colors adopted for the hands in different conditions. Likewise, the use of dashed and dotted lines is confused in the most important areas of these figures. Why not highlight the first 250 ms on a separate graph?

Finally, the colors adopted by experiment in figure 9 should not be the same colors adopted for each hand in the other figures.
