# Supplementary material for: Catch the star! Spatial information activates the manual motor system
Source: PLoS One. 2022 Jul 8;17(7):e0262510. doi: 10.1371/journal.pone.0262510 (PMC9269453; doi:10.1371/journal.pone.0262510)
Supplement: Attachment — Submitted filename: Response to Reviewer.docx [file pone.0262510.s004.docx]

**Reviewer #1:**

R1 – Although you have decided not to include the no-word stimuli results in the manuscript, you have decided to include its description. There is no problem about not having a hypothesis validated by the results, but it is not interesting that the data are not presented because they do not agree with that given hypothesis. I suggest exposing such data and discussing this topic in the manuscript - the hypothesis, the results, and their implication in the study - or removing this detail from the description of the experiment.

***REPLY: Thank you for this recommendation. I updated the manuscript accordingly:***

***Lines 376-378: “Symbol arrays were included to investigate potential lexicality effects, i.e., the difference in signal between word and non-word stimuli found, for example, in EEG at 150-250 ms after stimulus onset (Pulvermüller et al., 2009).”***

***Fig. 9A: I plotted a separate line for no-go trials with symbol arrays.***

***Lines 402-404: “Note that the pattern for symbol arrays closely resembles that for words, especially in the critical time window around 200 ms. The only difference between the two forces is the larger peak for symbol arrays around 650 ms.”***

***Lines 418-421: “As before, a cluster permutation analysis was used for exploratory purposes. First, Hand (right / left) and Lexicality (word / symbol array) as within-variables and their interaction were submitted. The analysis did not reveal any significant effects (all p-values > .72). This result indicates that lexicality is not reflected in the force signal; trials with symbol arrays were excluded from all further analyses.”***

***Lines 619-624 (Discussion): “An additional finding in Experiment 3 is that lexicality, i.e., the distinction between word and non-word stimuli, previously found to influence the EEG signal at 150-250 ms after stimulus onset (Pulvermüller et al., 2009), is not reflected in grip force signal. Unlike in most previous studies on lexical processing, meaningless symbol arrays (§@#$%) were used in the present study*** ***instead of pseudowords. Future research should consider using pseudowords built according to phonetic and morphological regularities of the language under investigation to confirm the present finding.”***

***I also updated the OSF materials by adding two scripts for interested readers:***

- ***Experiment 3: Words / 04 processing scripts / 011_cluster permutation_prepare_lexicality.R***
- ***Experiment 3: Words / 04 processing scripts / 012_cluster_permutation_analysis_lexicality.R***
